# Efficacy of biomechanics-based core muscle training in patients with postpartum stress urinary incontinence

**DOI:** 10.1097/MD.0000000000047763

**Published:** 2026-02-20

**Authors:** Xiaodan Yang, Xiaoyu Wu, Xiaoyan Su, Ping Li, Xiaoyun Wang

**Affiliations:** a Department of Pelvic Floor Rehabilitation, Shijiazhuang Maternal and Child Health Hospital, Shijiazhuang, Hebei, China.

**Keywords:** biomechanics-based core muscle training, modified Oxford scale, pelvic floor muscle training, postpartum stress urinary incontinence, rehabilitation outcomes

## Abstract

Postpartum stress urinary incontinence (SUI) is a frequent pelvic floor disorder caused by pregnancy- and delivery-related neuromuscular injury, leading to urine leakage during exertion. This study evaluated the efficacy and safety of combining biomechanics-based core training with standard PFMT in postpartum women with SUI. This retrospective study included 178 patients with postpartum SUI treated between May 2021 and May 2025. Based on the rehabilitation protocol, patients were divided into a control group receiving conventional PFMT and an observation group receiving additional biomechanics-based core training. Primary outcomes were changes in the 1-hour pad test and the International Consultation on Incontinence Questionnaire-Urinary Incontinence Short Form (ICIQ-UI SF). Secondary measures included pelvic floor strength assessed by the modified Oxford scale and safety evaluation. Statistical comparisons were performed using paired and independent-samples *t*-tests and chi-square tests. Both groups showed significant posttreatment improvement, with greater benefits in the observation group. The 1-hour pad test decreased from 17.9 ± 7.1 g to 8.7 ± 5.9 g versus 18.7 ± 7.5 g to 12.9 ± 6.5 g in controls (*P* < .001). ICIQ-UI SF scores declined by 6.8 versus 4.1 points (*P* < .001). The rate of ≥50% urine loss reduction was higher in the observation group (70.0% vs 46.6%; RR = 1.50, 95% CI 1.16–1.95; *P* = .002). Modified Oxford scale improvement was also greater (+1.60 vs +0.90; *P* < .001). No serious adverse events occurred. Biomechanics-based core training significantly improved urinary continence, pelvic floor strength, and quality of life without increasing adverse events, suggesting it is a safe and effective adjunct to conventional PFMT for postpartum rehabilitation.

## 1. Introduction

Postpartum stress urinary incontinence (SUI) represents one of the most prevalent pelvic floor disorders affecting women after childbirth, characterized by involuntary leakage of urine during physical exertion, coughing, or sneezing. It is primarily attributed to damage or dysfunction of the pelvic floor support system caused by pregnancy, vaginal delivery, and hormonal fluctuations. Epidemiological surveys indicate that approximately 20% to 35% of women experience urinary leakage in the early postpartum period, and up to one-third continue to exhibit symptoms beyond 6 months after delivery. Persistent postpartum SUI negatively influences quality of life, psychosocial well-being, and sexual health, making early and effective rehabilitation interventions a clinical priority.^[1,2]^ The pathophysiology of postpartum SUI involves weakening of the pelvic floor muscles and connective tissues, increased urethral mobility, and decreased urethral closure pressure. In addition to local muscle impairment, biomechanical studies suggest that altered coordination between the diaphragm, transversus abdominis, multifidus, and pelvic floor muscles may further impair pressure regulation within the abdominal cavity.^[3]^ Consequently, pelvic floor muscle training (PFMT) has long been recognized as the cornerstone of conservative management for postpartum SUI, with extensive evidence demonstrating its efficacy in improving urethral support and reducing urinary leakage.^[4,5]^ However, despite its benefits, the response to conventional PFMT remains heterogeneous, often limited by inadequate activation of deep core muscles, poor compliance, and nonphysiological recruitment patterns that fail to restore lumbopelvic stability.

Biomechanics-based core muscle training has recently emerged as a promising adjunctive strategy aimed at restoring integrated neuromuscular control across the trunk and pelvic floor. This approach emphasizes synchronized activation of the transversus abdominis, pelvic diaphragm, and deep spinal stabilizers to enhance intra-abdominal pressure modulation and reinforce pelvic organ support.^[6]^ By focusing on the mechanical interplay between the abdominal wall and pelvic floor, this method aims to achieve more physiologic continence mechanisms than isolated pelvic floor contraction. Preliminary studies suggest that combined core stabilization and PFMT interventions may yield superior improvements in muscle strength, urinary leakage reduction, and functional outcomes compared with traditional rehabilitation alone. Nevertheless, high-quality clinical data specifically addressing the efficacy and safety of biomechanics-based core training in postpartum women remain limited.^[7,8]^

Given this background, the present study aimed to evaluate the clinical efficacy and safety of biomechanics-based core muscle training combined with conventional pelvic floor rehabilitation in patients with postpartum SUI. The primary outcomes included objective urine leakage reduction (1-hour pad test) and subjective symptom improvement assessed by the International Consultation on Incontinence Questionnaire-Urinary Incontinence Short Form (ICIQ-UI SF). Secondary outcomes involved assessment of pelvic floor muscle strength using the modified Oxford scale (MOS), quality of life changes measured by validated questionnaires, and treatment-related adverse events. We hypothesized that the integrated biomechanics-based approach would provide greater improvements in urinary continence and pelvic floor function than conventional rehabilitation alone while maintaining an acceptable safety profile. Through this investigation, we sought to contribute robust clinical evidence supporting the application of a biomechanics-guided rehabilitation framework for postpartum SUI, offering both mechanistic and practical implications for early postpartum recovery and the optimization of women’s pelvic health care.

## 2. Materials and methods

### 2.1. Study design

This was a retrospective cohort study based on medical records of postpartum women with SUI treated at our institution between May 2021 and May 2025. All patients received rehabilitation interventions as part of routine clinical practice. The rehabilitation protocols, grouping, and follow-up assessments were determined by clinical decisions rather than by a predefined research protocol. Data were subsequently extracted and analyzed retrospectively for research purposes. No prospective randomization or intervention assignment was performed. Eligible participants met the following inclusion criteria: age between 20 and 45 years; clinical diagnosis of postpartum SUI confirmed by medical history, physical examination, and urodynamic testing in accordance with the International Continence Society (ICS) criteria; onset of symptoms within 12 months after delivery; and complete clinical data and regular follow-up records. Exclusion criteria included: urinary incontinence caused by urinary tract infection, neurological disease, pelvic organ prolapse, or urethral injury; a history of pelvic floor surgery or pelvic malignancy; current pregnancy or lactation; and inability to cooperate with training or incomplete treatment course. According to the therapeutic regimen received, patients were divided into 2 groups: the control group, which received conventional pelvic floor rehabilitation therapy, and the observation group, which received biomechanics-based core muscle training in addition to conventional therapy. All treatments were administered under the supervision of professional physiotherapists following standardized protocols. The study was conducted in accordance with the ethical principles outlined in the Declaration of Helsinki and was approved by the Institutional Medical Ethics Committee. Written informed consent was obtained from all participants prior to inclusion in the study.

### 2.2. Clinical diagnosis of postpartum stress urinary incontinence

The clinical diagnosis of postpartum SUI was established according to the diagnostic criteria recommended by the ICS and relevant Chinese Urology and Obstetrics and Gynecology guidelines. A definitive diagnosis required the presence of characteristic symptoms and confirmatory clinical findings, including the following:

1. Typical symptoms: Involuntary leakage of urine during physical exertion, coughing, sneezing, or other activities that increase intra-abdominal pressure, occurring after childbirth and persisting for at least 4 weeks postpartum.
2. Physical examination: Observation of urine leakage during the Valsalva maneuver or coughing in the lithotomy position with a comfortably filled bladder; no evidence of pelvic organ prolapse greater than stage II according to the POP-Q system.
3. Urodynamic evaluation: Demonstration of urine leakage coinciding with increased intra-abdominal pressure and without detrusor overactivity on cystometric testing, confirming stress-type incontinence.
4. Exclusion criteria for other causes: Absence of urinary tract infection, overflow incontinence, urge incontinence due to detrusor overactivity, or neurological disorders affecting bladder control.

Only patients who fulfilled all of the above criteria were confirmed to have postpartum SUI and were included in the study.

All rehabilitation interventions were delivered by the same pelvic floor rehabilitation team. Therapists involved in both groups had comparable professional qualifications and clinical experience and followed standardized training protocols. Treatment procedures were conducted according to unified operational guidelines to minimize performance bias.

### 2.3. Treatment protocol

All participants received standardized postpartum pelvic floor rehabilitation under the guidance of certified physiotherapists. Based on the treatment modality, patients were assigned to the control group or the observation group.

Control group: Patients in the control group underwent conventional pelvic floor rehabilitation, which included PFMT, bladder training, and daily lifestyle education. PFMT was performed using the Kegel method, involving repeated voluntary contractions and relaxations of the pelvic floor muscles for 10 seconds each, followed by a 10-second rest, 30 repetitions per session, twice daily. Bladder training emphasized timed voiding and avoidance of excessive intra-abdominal pressure. Participants were also instructed on weight control, perineal hygiene, and appropriate postpartum physical activity.

Observation group: In addition to the conventional rehabilitation described above, patients in the observation group received biomechanics-based core muscle training aimed at improving the coordinated stability of the pelvic floor and core trunk musculature. The training protocol emphasized activation of the transversus abdominis, multifidus, diaphragm, and pelvic floor muscles to enhance intra-abdominal pressure regulation and pelvic support. Exercises were conducted under real-time ultrasound or surface electromyographic biofeedback to ensure correct muscle activation patterns. The training program consisted of 3 phases – Initial initial phase (weeks 1–2): low-intensity static activation of the transversus abdominis and pelvic floor muscles in supine and bridge positions; Intermediate phase (weeks 3–6): progressive dynamic coordination exercises combining breathing control with trunk and pelvic stabilization; Advanced phase (weeks 7–12): functional core strengthening integrated into upright and daily activity movements, such as squats and balance training.

Each session lasted 30 to 40 minutes, performed 3 times per week for 12 consecutive weeks, with gradual intensity progression according to individual tolerance. Adherence was monitored, and incorrect movements were corrected under professional supervision.

### 2.4. Data collection

Two independent investigators extracted the data using a standardized electronic form to ensure accuracy and completeness, and any inconsistencies were resolved through consensus with a third reviewer. Baseline demographic and obstetric information included age, body mass index, mode of delivery (vaginal or cesarean), parity (primiparous or multiparous), interval from delivery to treatment initiation, and symptom duration. Baseline clinical data consisted of frequency of urinary leakage episodes per day, 1-hour pad test results (g), International Consultation on Incontinence Questionnaire-Urinary Incontinence Short Form (ICIQ-UI SF) scores, and MOS grades assessing pelvic floor muscle strength.

Throughout the 12-week rehabilitation period, adherence records, session frequency, and duration of each training session were documented. After completion of treatment, the same measurements were repeated to assess changes in objective leakage, symptom severity, and pelvic floor function. Quality of life was assessed pre- and post-intervention using 2 freely available validated instruments: the Incontinence Impact Questionnaire-Short Form (IIQ-7) and the Pelvic Floor Impact Questionnaire-Short Form (PFIQ-7). All adverse events (AEs) were systematically documented, including muscle soreness, perineal discomfort, fatigue, and low back pain. For each AE, the type, severity, duration, and clinical outcome were recorded. Serious adverse events (SAEs), defined as those resulting in hospitalization, permanent disability, or life-threatening conditions, were absent in both groups, confirming the safety of the interventions. To maintain data integrity. Pelvic floor muscle strength assessed by the MOS was evaluated by the same experienced therapist throughout the study period. Prior to assessment, evaluators underwent standardized training to ensure consistency and reliability. The assessor was not involved in treatment delivery.

### 2.5. Statistical analysis

All statistical analyses were performed using SPSS version 26.0 (IBM Corp., Armonk) and GraphPad Prism version 9.0 (GraphPad Software, San Diego). Continuous variables were first tested for normality using the Shapiro–Wilk test. Normally distributed data were expressed as mean ± standard deviation, while non-normally distributed data were presented as median (interquartile range, IQR). For baseline comparisons between the control and observation groups, independent-samples *t*-tests were used for continuous variables and Pearson’s chi-square test for categorical variables. Within-group pre- and posttreatment comparisons were analyzed using paired-samples *t*-tests for continuous variables. Between-group differences in treatment-related changes were assessed using independent-samples *t*-tests with 95% confidence intervals (CIs). All statistical tests were 2-tailed, and a *P* value < 0.05 was considered to indicate statistical significance.

## 3. Results

### 3.1. Baseline characteristics

At baseline, groups were comparable in age (31.8 ± 4.2 vs 31.5 ± 4.5 years; *t* = 0.460, *P* = .646) and body mass index (24.1 ± 3.2 vs 24.5 ± 3.1 kg/m^2^; *t* = –0.847, *P* = .397). Distribution of delivery mode (vaginal 62.5% vs 66.7%; χ^2^ = 0.338, *P* = .845) and parity (primiparous 59.1% vs 62.2%; χ^2^ = 0.183, *P* = .913) did not differ. The interval from delivery to treatment initiation (5.3 ± 1.9 vs 5.1 ± 1.8 months; *t* = 0.721, *P* = .471) and symptom duration (3.2 ± 1.4 vs 3.0 ± 1.5 months; *t* = 0.920, *P* = .358) were similar. Baseline incontinence severity was also comparable, as reflected by leakage frequency (3.8 ± 1.2 vs 3.6 ± 1.1 episodes/day; *t* = 1.158, *P* = .247) and 1-hour pad test loss (18.7 ± 7.5 vs 17.9 ± 7.1 g; *t* = 0.731, *P* = .465). These findings indicate balanced baseline characteristics between groups (Table 1).

**Table1:** Demographic and clinical characteristic.

Variable	Control (n = 88)	Observation (n = 90)	Test statistic	*P* value
Age, yr, mean ± SD	31.8 ± 4.2	31.5 ± 4.5	*t* = 0.460	.646
BMI, kg/m^2^, mean ± SD	24.1 ± 3.2	24.5 ± 3.1	*t* = –0.847	.397
Mode of delivery, n (%)			χ^2^ = 0.338	.845
Vaginal	55 (62.5)	60 (66.7)		
Cesarean	33 (37.5)	30 (33.3)		
Parity, n (%)			χ^2^ = 0.183	.913
Primiparous	52 (59.1)	56 (62.2)		
Multiparous	36 (40.9)	34 (37.8)		
Time from delivery to treatment, months, mean ± SD	5.3 ± 1.9	5.1 ± 1.8	*t* = 0.721	.471
Duration of SUI symptoms, months, mean ± SD	3.2 ± 1.4	3.0 ± 1.5	*t* = 0.920	.358
Leakage episodes per day, mean ± SD	3.8 ± 1.2	3.6 ± 1.1	*t* = 1.158	.247
1-hour pad test, g, mean ± SD	18.7 ± 7.5	17.9 ± 7.1	*t* = 0.731	.465

BMI = body mass index, SD = standard deviation, SUI = stress urinary incontinence.

### 3.2. Improvement in urinary incontinence severity

After 12 weeks of treatment, both groups showed improvement, but the observation group achieved significantly greater reductions. The 1-hour pad test decreased from 17.9 ± 7.1 g to 8.7 ± 5.9 g in the observation group versus 18.7 ± 7.5 g to 12.9 ± 6.5 g in the control group (between-group difference = −3.40 g, 95% CI −4.71 to −2.09; *t* = −5.15; *P* < .001). For ICIQ-UI SF, mean scores declined from 13.0 ± 3.0 to 6.2 ± 2.7 in the observation group and from 13.2 ± 3.1 to 9.1 ± 3.0 in the control group (difference = −2.70 points, 95% CI −3.37 to −2.03; *t* = −8.00; *P* < .001). Responder analysis (≥50% reduction in urine loss) showed higher improvement rates in the observation group (70.0% vs 46.6%; χ^2^ = 10.04; *P* = .002; RR = 1.50, 95% CI 1.16–1.95; Table 2).

**Table 2:** Combined summary of pre/post outcomes, within-group changes, and between-group comparisons for the 1-hour pad test and ICIQ-UI SF.

Outcome	Metric	Control (n = 88) mean ± SD or n (%)	Observation (n = 90) mean ± SD or n (%)	Between-group difference in change (Obs − Ctrl) Mean (95% CI)	Independent *t*, *P*
1-hour pad test, g	Baseline	18.7 ± 7.5	17.9 ± 7.1	–	–
	Posttreatment	12.9 ± 6.5	8.7 ± 5.9	−3.40 (−4.71 to −2.09)	−5.15, <.001
	≥50% reduction responders	41 (46.6%)	63 (70.0%)	RR 1.50 (1.16–1.95)	χ^2^ = 10.04, .002
ICIQ-UI SF, points	Baseline	13.2 ± 3.1	13.0 ± 3.0	–	–
	Posttreatment	9.1 ± 3.0	6.2 ± 2.7	−2.70 (−3.37 to −2.03)	−8.00, <.001

CI = confidence interval, Ctrl = control group, ICIQ-UI SF = International Consultation on Incontinence Questionnaire–Urinary Incontinence Short Form, Obs = observation group, RR = risk ratio, SD = standard deviation.

### 3.3. Pelvic floor muscle function

At baseline, MOS scores were comparable between groups (both 2.2 ± 0.8). After treatment, the observation group demonstrated significantly higher pelvic floor muscle strength (3.8 ± 0.9 vs 3.1 ± 0.9), with a mean posttreatment difference of 0.70 points (95% CI 0.43–0.97; *t* = 5.19; *P* < .001). The improvement from baseline was also greater in the observation group (+1.60 vs +0.90; difference 0.70, 95% CI 0.49–0.91; *t* = 6.67; *P* < .001). The posttreatment grade distribution differed significantly between groups (χ^2^ = 13.72, *P* = .008), indicating that more patients in the observation group progressed to higher MOS grades (IV–V). The rate of ≥1-grade improvement was 83.3% in the observation group versus 63.6% in controls (χ^2^ = 8.88; *P* = .003; RR = 1.31, 95% CI 1.08–1.60), confirming a superior enhancement of pelvic floor strength with biomechanics-based core muscle training (Table 3).

**Table 3:** MOS outcomes: pre/post means, grade distributions, improvement rates, and split between-group comparisons.

Outcome	Metric	Control (n = 88)	Observation (n = 90)	Between-group effect estimate (Obs − Ctrl) [95% CI]	Test statistic, *P*
MOS score (1–5)	Baseline mean ± SD	2.2 ± 0.8	2.2 ± 0.8	–	–
	Posttreatment mean ± SD	3.1 ± 0.9	3.8 ± 0.9	0.70 [0.43, 0.97]	*t* = 5.19, *P* < .001
	Change (post − baseline), mean [95% CI]	0.90 [0.75, 1.05]	1.60 [1.45, 1.75]	0.70 [0.49, 0.91]	*t* = 6.67, *P* < .001
MOS grade distribution (I–V), n (%)	Baseline	I 22 (25.0) • II 38 (43.2) • III 20 (22.7) • IV 8 (9.1) • V 0 (0)	I 22 (24.4) • II 36 (40.0) • III 22 (24.4) • IV 10 (11.1) • V 0 (0)	–	–
	Posttreatment	I 10 (11.4) • II 30 (34.1) • III 30 (34.1) • IV 16 (18.2) • V 2 (2.3)	I 4 (4.4) • II 15 (16.7) • III 35 (38.9) • IV 28 (31.1) • V 8 (8.9)	global distribution	χ^2^ = 13.72, *P* = .008
Improvement rate	≥1-grade increase, n/N (%)	56/88 (63.6%)	75/90 (83.3%)	RR 1.31 [1.08, 1.60]	χ^2^ = 8.88, *P* = .003

CI = confidence interval, Ctrl = control group, MOS = modified Oxford scale, Obs = observation group, RR = risk ratio, SD = standard deviation.

### 3.4. Adverse events

Mild, self-limited AEs were observed in 20.5% of controls and 24.4% of the observation group (RR 1.19, 95% CI 0.69–2.06; *P* = .52). The most frequent symptoms were muscle soreness (11.4% vs 20.0%; RR 1.75, 95% CI 0.86–3.59; *P* = .11) and perineal discomfort (9.1% vs 13.3%; RR 1.46, 95% CI 0.63–3.40; *P* = .35). Rates of low back discomfort and fatigue were low and comparable between groups (all *P* > .05). Training interruption due to AEs was rare (2.3% vs 3.3%; *P* = .68). No SAEs occurred. Overall, biomechanics-based core muscle training demonstrated a favorable safety profile comparable to conventional rehabilitation (Table 4).

**Table 4:** Treatment-related adverse events.

AE	Control (n = 88)	Observation (n = 90)	RR (Obs/Ctrl) [95% CI]	*P* value
Any treatment-related AE	18 (20.5%)	22 (24.4%)	1.19 [0.69–2.06]	.52
Muscle soreness	10 (11.4%)	18 (20.0%)	1.75 [0.86–3.59]	.11
Perineal tension/discomfort	8 (9.1%)	12 (13.3%)	1.46 [0.63–3.40]	.35
Low back discomfort	6 (6.8%)	8 (8.9%)	1.30 [0.47–3.61]	.60
Fatigue	7 (8.0%)	9 (10.0%)	1.26 [0.49–3.23]	.62
Training interruption due to AE	2 (2.3%)	3 (3.3%)	1.47 [0.25–8.58]	.68
Serious adverse events (SAE)	0 (0%)	0 (0%)	–	–

AE = adverse event, CI = confidence interval, RR = risk ratio, SAE = serious adverse event.

## 4. Discussion

This retrospective analysis indicates that adding biomechanics-based core muscle training to conventional pelvic floor rehabilitation is associated with greater reductions in leakage volume and symptom burden in postpartum SUI than conventional therapy alone. After 12 weeks, the observation group demonstrated larger decreases in 1-hour pad test values and ICIQ-UI SF scores, higher responder rates for ≥50% reduction in urine loss, and greater gains in pelvic floor muscle strength measured by the MOS. Safety profiles were comparable between groups, and no serious adverse events were observed. Collectively, the findings support an integrated approach targeting both pelvic floor activation and core stabilization to improve continence outcomes in the postpartum period.

The magnitude and consistency of improvements across objective (pad test), patient-reported (ICIQ-UI SF), and clinician-rated (MOS) measures suggest that synergy between transversus abdominis activation, diaphragmatic control, and pelvic floor contraction contributes to continence recovery. SUI is characterized by a mismatch between intra-abdominal pressure surges and urethral support. Coordinated activation of deep trunk muscles can modulate intra-abdominal pressure and augment urethral closure forces via fascial and myofascial connections with the levator ani, thereby reducing leakage episodes during exertion.^[9,10]^ The higher proportion of patients achieving MOS grade IV–V after the integrated intervention implies that neuromuscular recruitment patterns improved alongside strength, not merely short-term compensatory strategies. The absence of a safety signal, with only mild, self-limited symptoms and similar event rates between groups, further indicates that the additional core component is tolerable within supervised rehabilitation. The responder analysis complements mean differences by quantifying clinically interpretable benefit. In this cohort, the number needed to treat to achieve a ≥50% pad-test reduction approximates 4, derived from absolute risk difference between groups, which is aligned with an effect of practical relevance in postpartum rehabilitation services.^[11,12]^ The concordant decline in IIQ-7 and PFIQ-7 scores (quality of life instruments) reinforces that symptom changes translated into functional improvement.

Contemporary guidelines continue to recommend PFMT as first-line therapy for SUI, including postpartum populations, emphasizing supervised programs and validated outcome measures. The 7th International Consultation on Incontinence and recent ICS standards reaffirm PFMT’s central role and the use of instruments such as ICIQ-UI SF for responsive outcome assessment. Our results are congruent with these recommendations and extend them by explicitly incorporating a biomechanics-oriented core module. In randomized and quasi-experimental studies from the last 3 years, adding adjunctive modalities to PFMT enhanced outcomes relative to PFMT alone.^[13,14]^ A 2024 randomized clinical trial showed that pressure-mediated biofeedback combined with PFMT improved SUI symptom scores and objective measures more than PFMT alone, underscoring the value of targeted, feedback-guided activation of pelvic musculature. While that intervention did not explicitly train the trunk, it supports the general concept that augmenting PFMT with targeted components yields incremental benefit; our data suggest that a core-focused adjunct provides a similar or greater effect on leakage reduction.^[15]^

Evidence specific to core or transversus abdominis (TrA) training in women with SUI has grown. Studies comparing PFMT against core stabilization report superior or additive effects on symptom severity and functional measures when TrA activation is emphasized, likely through improved lumbopelvic stability and intra-abdominal pressure control. Reports in 2024 demonstrated that combining PFMT with TrA or core exercises yielded greater improvements in incontinence parameters than PFMT alone. Our findings align with these data and provide postpartum-specific confirmation using both objective pad testing and clinician-rated MOS. Recent evidence syntheses also maintain that postpartum PFMT reduces the odds of urinary incontinence and improves symptoms, though they frequently call for standardized protocols and better specification of adjuncts. The present study answers part of this gap by specifying a biomechanics-based core protocol layered onto standard PFMT and showing superiority across multiple endpoints.^[16]^

For clinical practice, integrating biomechanics-based core training into standard postpartum PFMT may increase the probability and magnitude of continence recovery within a 12-week schedule without compromising safety. Programs should incorporate supervised TrA-focused activation, breathing control, and progressive trunk stabilization alongside conventional PFMT, ideally with technique verification and adherence monitoring. Adoption of responsive measures (ICIQ-UI SF, pad test, MOS) enables objective tracking and early course correction. Clinics with established PFMT infrastructure can implement this integrated protocol with minimal additional equipment, using structured progressions and clear return-to-activity guidance to support postpartum rehabilitation pathways. Strengths include a clearly defined postpartum SUI cohort with standardized diagnostic criteria; multimodal outcome assessment incorporating objective leakage, validated symptoms, and graded strength; an explicit, progressive biomechanics-based core protocol added to conventional PFMT; and responder analyses and confidence intervals that facilitate clinical interpretation. The safety assessment captured both event types and training interruption, supporting feasibility.

Limitations merit careful consideration. First, the retrospective design introduces susceptibility to selection bias and unmeasured confounding; causal inference is therefore limited compared with prospective randomization. Second, the intervention lasted 12 weeks; durability beyond this interval is unknown, and posttreatment maintenance strategies were not evaluated. Third, adherence was supervised but not objectively quantified outside scheduled sessions, and dose-response relationships remain undefined. Fourth, the study was single-center, limiting generalizability across diverse obstetric practices and cultural rehabilitation norms. Fifth, MOS, though pragmatic and widely used, remains an ordinal, examiner-dependent scale; future work should complement MOS with instrumented measures or biofeedback-derived strength indices when feasible. Finally, although the sample size supported detection of clinically relevant between-group differences, subgroup effects (e.g., parity, delivery mode, perineal trauma) were not powered for formal interaction testing. Although standardized training protocols were applied, potential performance bias related to therapist delivery cannot be fully excluded. Third, patients opting for biomechanics-based core training may have higher motivation and compliance, which could introduce selection bias. These factors may have influenced treatment outcomes and should be considered when interpreting the results. Prospective randomized controlled trials are warranted to further validate these findings.

## 5. Conclusions

Biomechanics-based core muscle training significantly enhanced pelvic floor strength and reduced urinary leakage in postpartum women with SUI compared with conventional rehabilitation alone. The intervention improved both objective and subjective outcomes without increasing adverse events. These findings support incorporating biomechanics-based core training into postpartum pelvic floor rehabilitation to achieve greater continence recovery and functional improvement with a favorable safety profile.

## Author contributions

**Conceptualization:** Xiaodan Yang, Xiaoyu Wu, Xiaoyan Su, Ping Li, Xiaoyun Wang.

**Data curation:** Xiaodan Yang, Xiaoyu Wu, Xiaoyan Su, Ping Li, Xiaoyun Wang.

**Formal analysis:** Xiaodan Yang, Xiaoyu Wu, Xiaoyan Su, Ping Li, Xiaoyun Wang.

**Funding acquisition:** Xiaodan Yang, Xiaoyun Wang.

**Investigation:** Xiaodan Yang, Xiaoyun Wang.

**Writing – original draft:** Xiaodan Yang, Xiaoyun Wang.

**Writing – review & editing:** Xiaodan Yang, Xiaoyu Wu, Xiaoyun Wang.
